# New Insights to Regulation of Fructose-1,6-bisphosphatase during Anoxia in Red-Eared Slider, *Trachemys scripta elegans*

**DOI:** 10.3390/biom11101548

**Published:** 2021-10-19

**Authors:** Aakriti Gupta, Anchal Varma, Kenneth B. Storey

**Affiliations:** Department of Biology, Carleton University, Ottawa, ON K1S 5B6, Canada; aakritigupta@cmail.carleton.ca (A.G.); anchalvarma@cmail.carleton.ca (A.V.)

**Keywords:** *Trachemys scripta elegans*, Fructose-1,6-bisphosphatse, enzyme regulation, gluconeogenesis, anoxia, metabolic rate depression, post-translational modification

## Abstract

The red-eared slider (*Trachemys scripta elegans*) undergoes numerous changes to its physiological and metabolic processes to survive without oxygen. During anoxic conditions, its metabolic rate drops drastically to minimize energy requirements. The alterations in the central metabolic pathways are often accomplished by the regulation of key enzymes. The regulation of one such enzyme, fructose-1,6-bisphosphatase (FBPase; EC 3.1.3.11), was characterized in the present study during anoxia in liver. FBPase is a crucial enzyme of gluconeogenesis. The FBPase was purified from liver tissue in both control and anoxic conditions and subsequently assayed to determine the kinetic parameters of the enzyme. The study revealed the relative degree of post-translational modifications in the FBPase from control and anoxic turtles. Further, this study demonstrated a significant decrease in the maximal activity in anoxic FBPase and decreased sensitivity to its substrate Fructose-1,6-bisphosphate (FBP) when compared to the control. Immunoblotting demonstrated increased threonine phosphorylation (~1.4-fold) in the anoxic FBPase. Taken together, these results suggest that the phosphorylation of liver FBPase is an important step in suppressing FBPase activity, ultimately leading to the inhibition of gluconeogenesis in the liver of the red-eared slider during anaerobic conditions.

## 1. Introduction

The red-eared slider turtle, *Trachemys scripta elegans* (*T. s. elegans*), is one of the best-studied models of facultative anaerobiosis among vertebrate species. These turtles have the ability to survive for several weeks in oxygen-deprived conditions when hibernating in cold water at temperatures of 0–5 °C [1]. In contrast to mammals that show extreme sensitivity to brief anoxia exposures, turtles have physiological and biochemical adaptations to endure prolonged anoxia, including high glycogen reserves, antioxidant defenses [2], metabolic rate depression, and an ability to buffer high concentrations of lactic acid generated by anaerobic glycolysis by sequestering lactate into the shell [3]. The metabolism is reduced to about 10–20% of the aerobic values [4], causing tissues to switch to a reliance on glycolysis alone for ATP production. Maintaining cellular energetics is one of the major concerns under anoxic conditions. This is because the conversion of glucose to lactate via glycolysis yields only 2 ATP per glucose molecule catabolized compared with the full aerobic oxidation of glucose to CO_2_ and H_2_O, which yields 36 ATP per glucose [5]. Therefore, studies of the molecular mechanisms that support long-term anoxia tolerance are important for understanding this remarkable phenomenon.

One major contributor to anoxia tolerance is metabolic rate depression, a process that leads to the strong and coordinated suppression of most of the metabolic processes. The biochemical mechanisms contributing to metabolic arrest include changes in the enzyme specificity, post-transcriptional and post-translational modifications, and reduced rates of protein biosynthesis [5,6]. Studies on stress responsive changes in the activities, kinetic properties, and regulatory mechanisms of the enzymes involved in carbohydrate catabolism have provided insights into the metabolic adjustments used by turtles to suppress glycolysis under anoxic conditions [7,8,9]. These studies demonstrated organ-specific changes in the kinetics and activities of enzymes, including glycogen phosphorylase, phosphofructokinase, pyruvate kinase, lactate dehydrogenase, and fructose bisphosphate aldolase under anoxic conditions [8,9,10,11,12]. In addition, anoxia also triggered tissue-specific changes in the activities of many other metabolic enzymes in *T. s. elegans* [2]. These studies have documented that the global control of glycolytic and other enzymes is vital for turtles to adapt during oxygen-depriving conditions [13,14,15,16,17].

**Figure 1:**
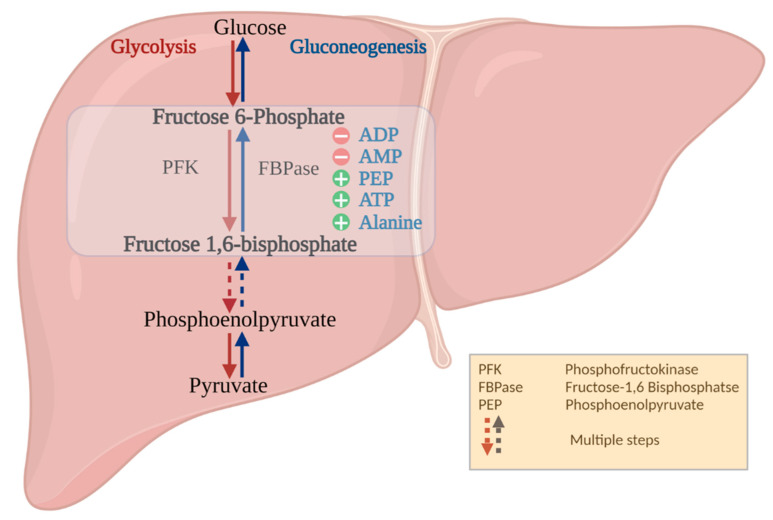
An overview of the allosteric regulation of FBPase on gluconeogenesis. The schematic diagram shows the regulatory mechanism of FBPase during conversion of FBP to Fructose 6-phosphate during gluconeogenesis. The diagram shows the activators and inhibitors for the enzyme. The blue arrows represent gluconeogenesis, and the red arrows represent glycolysis. The activators are represented as ‘+’ sign in green circle, and inhibitors are represented as ‘-’ sign in red circle. PFK: phosphofructokinase, FBPase: Fructose-1,6 bisphosphate, FBP: Fructose 1,6 bisphosphate, PEP: phosphoenolpyruvate. Credit “Created with BioRender.com”.

During anaerobiosis, turtles utilize large reserves of liver glycogen for energy production. Hepatic glycogenolysis acts as a source of glucose to other tissues during prolonged anoxic conditions [18]. Glucose can also be synthesized from noncarbohydrate precursors (such as amino acids) or during the clearance of accumulated lactate by a process called gluconeogenesis that occurs primarily in the liver. Limited work has been done with respect to the role that gluconeogenesis might play in fuel production in anoxia-tolerant animals, and further research in this area is warranted. Fructose-1,6-bisphosphatse (FBPase; EC 3.1.3.11) is a key regulatory enzyme in the gluconeogenic pathway and catalyzes the reverse reaction to that of phosphofructokinase (PFK) in glycolysis (Figure 1). Specifically, FBPase catalyzes the removal of a phosphate from Fructose-1,6-bisphosphate to form fructose-6-phosphate, in the presence of metal ions, magnesium or manganese. FBPase and PFK act as rate-limiting enzymes to control the gluconeogenic versus glycolytic pathways, respectively, resulting in the regulation of glucose homeostasis and energy metabolism [19]. FBPase is an allosteric enzyme (Figure 1). Its activity is stimulated by high ATP concentrations, whereas AMP and fructose 2,6-biphosphate are negative regulators [20]. The regulation of FBPase is also accomplished by reversible protein phosphorylation [21,22]. This strict regulatory network of FBPase and its importance in the gluconeogenic pathway makes it an intriguing enzyme to study in turtles that accumulate large amounts of lactate under anoxic conditions. The present investigation aims to explore the physical, kinetic, and regulatory properties of liver FBPase to determine its role in anoxia survival.

## 2. Materials and Methods

### 2.1. Animals

Adult red-eared sliders (*T. s. elegans*), 700–1500 g, were acquired from a local supplier (Ottawa, Ontario, Canada) and held at 5 ± 1 °C in large 50 L plastic tanks filled with dechlorinated tap water for 7–8 days before use. Control (normoxic) turtles were sampled from this condition. For anoxia exposure, turtles were transferred to large buckets containing water at 5 ± 1 °C that had been previously bubbled for 1 h with N_2_ gas via a port in the lid of the bucket. Two turtles were added per bucket, and a wire mesh was fitted into the bucket about 5 cm below the water surface so that turtles remained submerged throughout the 20 h experimental anoxia exposure. Bubbling with nitrogen gas was continued for 1 h after turtles were added and was reinitiated again during sampling of the animals. All animals were killed by decapitation and liver tissue was rapidly dissected out, frozen in liquid nitrogen, and stored at −80 °C until use. All animals were cared for in accordance with the guidelines of the Canadian Council on Animal Care, and experimental procedures had the prior approval of the Carleton University Animal Care Committee (protocol #106937, 11 June 2017).

### 2.2. Sample Preparation

Samples of liver tissue from control and 20 h anoxic turtles were rapidly weighed and homogenized in ice-cold homogenization buffer A (25 mM MES buffer, pH 6.25 mM NaF, 2.5 mM EDTA, 2.5 mM EGTA, 5 mM β-mercaptoethanol and 10% *v/v* glycerol) in the ratio 1:5 *w:v*. A few crystals of phenylmethylsulphonyl fluoride were added to the buffer immediately before the homogenization. Samples were homogenized using a Polytron homogenizer (Brinkmann Instruments, Westbury, NY, USA) followed by centrifugation at 13,500× *g* at 4 °C for 30 min. The supernatant was collected as crude homogenate and stored on ice.

### 2.3. FBPase Purification

FBPase was purified using a two-column procedure. A phosphocellulose column was prepared and equilibrated with 30 mL of buffer A. Supernatant was then loaded onto the column followed by washing with 40 mL of buffer A. FBPase then eluted using 30 mL of a 0 to 1 M KCl gradient containing 2.5 mM Fructose-1,6-bisphosphate (FBP) in buffer A. Fractions were collected using an automated fraction collector (Gilson Medical Electronics, Inc., Middletown, WI, USA). The highest activity fractions were pooled and loaded on a carboxymethyl (CM) cellulose column pre-equilibrated with buffer A. The column was then washed with 20 mL of buffer A and the enzyme was eluted using a 30 mL linear salt gradient of 0 to 1 M KCl. The fractions with highest FBPase activity were pooled and then analyzed by sodium dodecyl sulfate polyacrylamide gel electrophoresis (SDS–PAGE) to demonstrate purity of the FBPase preparation.

### 2.4. SDS PAGE and Coomassie Staining

The purity of FBPase was confirmed by running SDS–PAGE using a protocol described previously by Smolinski et. al. [23]. Samples of FBPase for SDS–PAGE were prepared using equal aliquots of purified enzyme and SDS loading buffer mixed (100 mM Tris buffer, pH 6.8, 4% *w:v* SDS, 20% *v:v* glycerol, 0.2% *w:v* bromophenol blue) with 10% *v:v* β-mercaptoethanol added fresh. Samples were boiled for 5 min, then cooled on ice and stored in a freezer at −20 °C. Subsequently, enzyme samples were loaded into wells of 10% gel and run adjacent to a low molecular weight ladder (Pink Plus Prestained Protein Ladder, Frogga Bio, Toronto, ON, Canada, catalog no. PM005–0500) for 55 min at 180 V. Silver staining was used following the procedure of Celis et. al. [24]. After the bands were clearly visible, the gel was imaged using Chemi-Genius Bio-Imaging system with GeneSnap software (Syngene, Frederick, MD, Canada). FBPase was further identified by excising the bands obtained from electrophoresis and sending them for mass spectrometry at the Proteomics Platform of the Quebec Genomics Center (Quebec, Boulevard Laurier, G1V 4G2, Canada).

### 2.5. FBPase Assay

FBPase was assayed by monitoring NADP+ reduction at 340 nm using a microplate reader (Multiskan Spectrum, Thermo Labsystems, Finland). Optimal assay conditions were 15 mM imidazole-HC1 buffer, pH 7.2, 0.1 mM Fructose-1,6-bisphosphate, 5 mM MgSO_4_, 0.2 mM NADP^+^, and 1 U each of phosphoglucoisomerase and glucose-6-phosphate dehydrogenase in a total volume of 200 μL. All assays were performed at 22 °C. K_m_ values were calculated using the above-mentioned assay conditions but with suboptimal concentrations of FBP (0.24 µM). I_50_ values for AMP and ADP and K_a_ values for alanine, phosphoenolpyruvate (PEP), and ATP.Mg (1:1) were also determined using similar assay methods.

Soluble protein concentrations were determined using the Coomassie blue dye-binding method with the BioRad Laboratories (Mississauga, ON, Canada) prepared reagent and bovine serum as the standard. Spectrophotometric quantification was at 595 nm.

### 2.6. Post-Translational Modification Analysis via Western Blotting

Post-translational modifications (PTMs) were assessed via immunoblotting. The protocol followed was as in Gupta and Storey, 2020 [25]. Following electrophoresis (as described above), proteins were transferred to a polyvinylidiene difluoride (PVDF) membrane by electroblotting at a constant current of 160 mA for 90 min under transfer buffer (25 mM Tris, pH 8.5, 192 mM glycine, 10% *v/v* methanol). After transfer, membranes were washed for 3 × 5 min using TBST (Tris-buffered saline with Tween-20): 20 mM Tris base pH 7.6, 150 mM NaCl, 0.05% *v/v* Tween-20). Following washing, membranes were probed with primary antibody, diluted 1:1000 *v/v* in TBST with overnight incubation at 4 °C on a rocker.

Polyclonal antibodies were used to detect: (a) serine phosphorylation (catalogue# 618100) (Invitrogen, Carlsbad, CA, USA) (b) threonine phosphorylation (catalog# 718200) (Invitrogen, Carlsbad, CA, USA) (c) tyrosine phosphorylation (catalog# 136600) (purchased from Invitrogen, Carlsbad, CA, USA); (d) ubiquitination (catalog# ab19247 Abcam, Cambridge, UK); and (e) SUMOylation 1 (a gift from the Clinical Investigations Section Stroke Branch, NINDS, Bethesda, MD, USA).

After incubation, membranes were washed for 3 × 5 min in TBST at room temperature and then incubated with secondary antibody (anti-rabbit IgG secondary antibody conjugated with horseradish peroxidase (catalog no. APA007P.2, Bioshop, Burlington, ON, Canada)) at 1:5000 *v:v* in TBST for 30 min. Membranes were again washed 3 times with TBST for 5 min per wash. Following the washes, immunoreactive bands on the membrane were detected using 600 µL each of luminol and hydrogen peroxide and imaged using the ChemiGenius Bioimaging System (Syngene, MD, USA). Membranes were subsequently stained with Coomassie blue (0.25% *w/v* Coomassie brilliant blue, 7.5% *v/v* acetic acid, and 50% methanol), and the summed intensities of a group of Coomassie-stained bands that were well separated from the band of interest and constant across all lanes were used to standardize the chemiluminescence of the immunobands of interest. Band intensities were quantified using GeneTools software (4.3.8.0, Syngene, Frederick, MD, USA).

### 2.7. In Silico FBPase 3D Homology Modelling

The amino acid sequence of red-eared slider turtle FBPase (Genbank XP_034629682.1) was used to predict the 3D structure of the enzyme using a homology modelling 3D protein structure prediction tool SWISS-MODEL (https://swissmodel.expasy.org/; accessed on 30 December 2020). Turtle FBPase was modelled against the X-ray crystal structure of human liver FBPase as it had the highest sequence coverage as compared with the turtle enzyme. Structural validations were performed using SWISS-MODEL, and the structural assessment tool was used to analyze QMEAN scores, Ramachandran plots, and MolProbity quality scores. Visualization of the FBPase structure in the presence of FBP and Mg was done using PyMOL Molecular Graphics System, Version 2.0 Schrödinger, LLC (DeLano Scientific LLC, CA, USA).

### 2.8. Quantification and Statistical Analysis

All enzyme assays were analyzed using a microplate analysis program (MPA), and enzyme kinetic parameters (K_m_, V_max_, I_50_, K_a_) were determined using a least squares regression computer program, Kinetics v. 3.5.1 [23]. Data for Western blots and kinetic parameters were analyzed using one-way analysis of variance (ANOVA), followed by Student’s *t*-test or Tukey’s post hoc test with *p* < 0.05 accepted as a significant difference.

## 3. Results

### 3.1. Purification of Fructose-1,6-bisphosphatase

**Figure 2:**
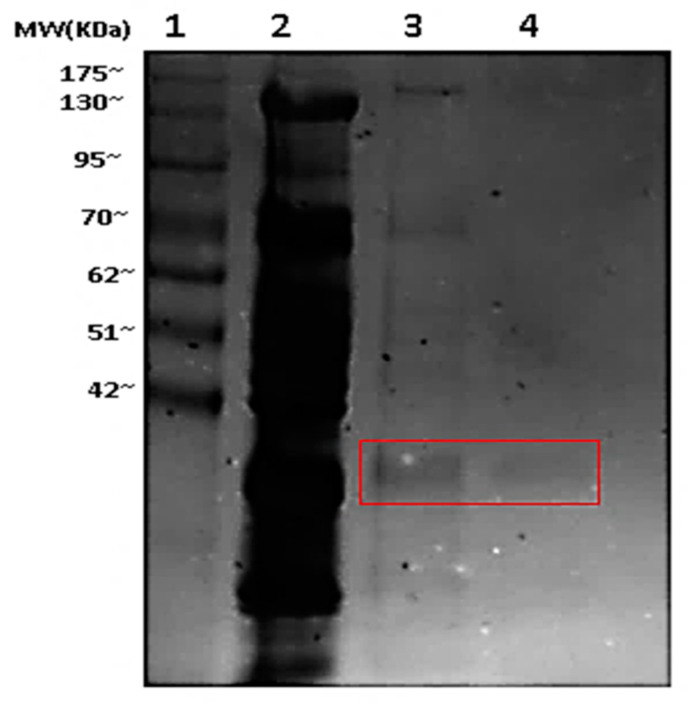
10% SDS–PAGE gel showing liver FBPase purification. Lanes are: (1) Molecular weight ladder (Froggabio); (2) crude homogenate; (3) pooled peak fractions after elution from phosphocellulose; (4) pooled peak fractions after elution from CM column.

The FBPase from the *T. s. elegans* liver was purified to homogeneity in a two-step chromatographic process. The first step, the elution of the FBPase from a phosphocellulose column, used a 0–1 M KCl linear gradient with 2.5 mM of FBP added. This was effective at removing many impurities and resulted in a 3.80-fold purification with 51.3% activity recovered (Table 1). The second step used a CM column and eluted FBPase using a 0–1 M KCl linear gradient, resulting in a final fold purification of 8.7 and a 24.8% yield of enzyme activity (Table 1). Using the SDS–PAGE followed by staining with silver demonstrated that the FBPase was purified to homogeneity. The purified FBPase was calculated to be ~37 kDa based on the electrophoretic mobility compared to the protein standards loaded (Figure 2).

### 3.2. Kinetic Properties of FBPase

The enzymatic parameters were measured using purified FBPase from the control and anoxia-exposed turtles. The V_max_ of the enzyme from the anoxic liver was significantly lower by 26% as compared with the control liver. The enzymes also differed in multiple kinetic parameters (Table 2). The K_m_ FBP of the FBPase increased significantly by 2.2-fold for the enzyme from the anoxic liver, rising from 26.9 µM for the control enzyme to 59.8 µM for the FBPase from the anoxic liver. The effects of the inhibitors on the relative activities of the control and anoxic liver FBPase were also determined (Table 2). There were significant decreases in the *I*_50_ values between the control and anoxic FBPase for both the ADP and AMP effects. The I_50_ for the ADP inhibition of anoxic liver FBPase (8.3 ± 0.6 µM) was significantly lower by 30% as compared to the control value (*I*_50_ = 11.8 ± 0.3 mM). Similarly, a 41% decrease compared to the control was observed for the *I*_50_ value for anoxic FBPase in the presence of AMP, with the control AMP *I*_50_ being 6.1 ± 0.1 µM, whereas the anoxic FBPase showed an AMP *I*_50_ of 3.6 ± 0.3 µM (Table 2).

ATP.Mg, PEP, and alanine were also investigated as activators of FBPase (Table 2). For ATP.Mg, the K_a_ value was significantly lower in the anoxia (25 ± 0.9 µM) as compared to that of the controls (144.5 ± 7.0 µM). By contrast, the K_a_ of the PEP was significantly higher in the anoxia (3.3± 0.2 µM) than in the controls (0.33 ± 0.09 µM). The activity of the anoxic enzyme in the presence of the PEP decreased to 0.3% compared to the control FBPase. The K_a_ for the L-alanine did not show any significant difference between the two conditions (Table 2).

### 3.3. PTM Western Blots

**Figure 3:**
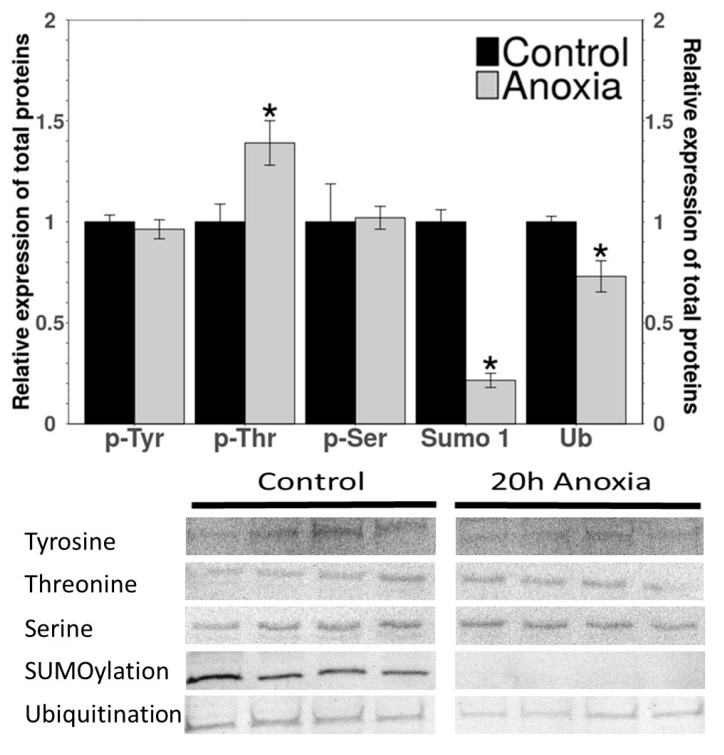
Western blot analysis to evaluate post-translational modifications in *T. s. elegans* liver FBPase from control versus anoxia conditions. Relative band intensity is defined as the quotient of the intensity of the chemiluminescent signal divided by the band intensity observed after staining with Coomassie brilliant blue. ‘*’ indicates that values for anoxic FBPase are significantly different from the control (*n* = 3–4) as determined by Student’s *t*-test *p* < 0.05.

The analysis of the relative levels of the PTMs was done using Western blotting (Figure 3). The phosphorylation of the threonine residues showed a 1.4-fold increase in the FBPase from anoxic liver as compared to the controls. However, the SUMOylation and ubiquitination showed a significant decrease to 73% and 22% of the control values, respectively (Figure 3).

### 3.4. Bioinformatics

**Figure 4:**
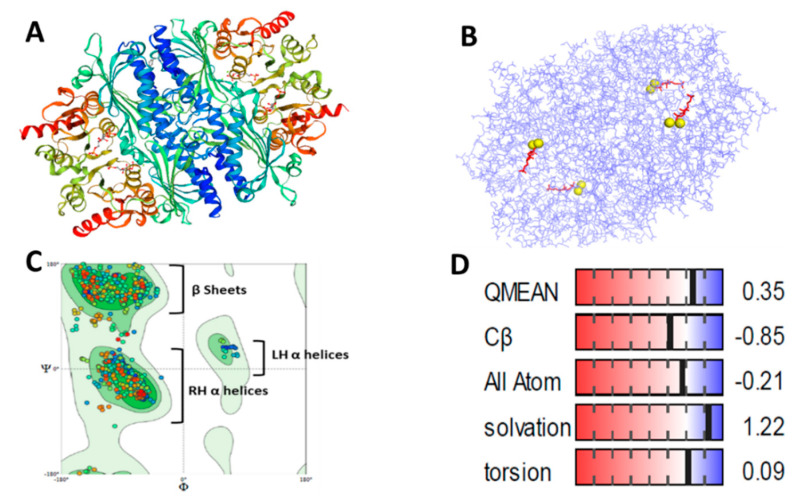
*T. s. elegans* FBPase 3D homology structure and structure assessments. (**A**) Predicted homology model of *T. s. elegans* FBPase generated using SWISS-MODEL with the *T. s. elegans* FBPase amino acid sequence (NCBI) and X-ray crystallized human liver FBPase as the structural template. Each chain of the tetramer is in a different color. (**B**) Visualization of the FBPase structure with FBP and Mg was done using PyMOL Molecular Graphics System, Version 2.0 Schrödinger, LLC. (**C**) Ramachandran plot of the amino acid torsional angles for *T. s. elegans* FBPase 3D homology model shows favorable β-sheet and α-helix angles. (**D**) QMEAN structure scores as determined by SWISSMODEL. All tests show numbers that are close to zero in the white area of the bar. Values close to or higher than zero indicate model scores that are expected or better than experimental structures of similar size.

A predicted 3D structure of the red-eared slider turtle FBPase was generated using the SWISS-MODEL. The primary sequence of the *T. s. elegans* enzyme was obtained from the NCBI (XP_034629682.1), and the crystal structure of human liver FBPase was used as the template to obtain a structural model of the turtle enzyme (Figure 4A). The visualization of the FBPase structure in the presence of the FBP substrate (red) and Mg (yellow) was done using Pymol and shows the position of these substrates at the active site (Figure 4B). The MolProbity structure-validation web service, a component of the SWISS-MODEL structure assessment tool, was used to provide an evaluation of the model quality at both the global and local protein level. A low global MolProbity score of 1.33 reflects that crystallographic resolution is at a good, expected quality. The generated Ramachandran plot was analyzed with MolProbity and showed a Ramachandran favored score of 95.62%, close to the ideal score of >98% (Figure 4C). The QMEAN score, a composite estimator of the geometrical properties at the global and local protein level, was calculated to be 0.35 (Figure 4D). This score considers the Cβ atoms interaction energy of −0.85, all-atom pairwise energy of −0.21, solvation energy of 1.22, and torsion angle energy of 0.09 (Figure 4D). Collectively, these physics and statistical knowledge-based modelling scores are all near zero, which indicates a model that is in the range of experimental models of the same size.

## 4. Discussion

A lack of oxygen is a major challenge to the survival of many animals. The red-eared slider, *T. s. elegans*, is an anoxia-tolerant species and can survive without oxygen for weeks during the winter when hibernating in cold water [1,26]. Turtles also experience oxygen deprivation during breath-hold diving in all seasons, and this escalates to prolonged periods of anoxia during overwintering in ice-locked ponds [27]. Under anoxic conditions, turtles rely on their glycogen reserves for energy and switch to lactic acid fermentation [18]. In addition, they lower their metabolic rate to only about 10% of the normal rate [9], making them model organisms to study the remodeling of the energy metabolism for extended periods of time [28]. Profound metabolic rate depression enables survival without oxygen for several months at cold temperatures. By rearranging key cellular processes, turtles can redirect the available fuel/energy resources to prioritize pro-survival processes that are important during anoxia. Considering that glycogen is metabolized under anoxic conditions as a fuel/energy source [7], it is likely that turtles pre-load their organs with high glycogen stores during summer/autumn feeding. Therefore, gluconeogenesis could be one of the active processes occurring under aerobic conditions. Gluconeogenesis is also important for the recovery of internal glycogen reserves when turtles transition back from prolonged anoxia to air-breathing. FBPase is the rate-limiting enzyme of gluconeogenesis. A study of its enzymatic mechanism would provide better insight into the metabolism restructuring by turtles under anoxic conditions. Indeed, the present study showed that liver FBPase from the aerobic control and anoxic *T. s. elegans* displayed markedly different properties, and further investigation linked these differences in enzyme properties with the anoxia-responsive covalent modification of the enzyme.

The maximum activity of the FBPase (expressed as U/mg protein) purified from the liver of anoxic turtles was reduced significantly as compared to that of the control enzyme from aerobic turtles. This lower FBPase V*_max_* suggests that a stable modification of the enzyme occurred in response to anoxia, potentially reducing the FBPase function under anoxic cellular conditions where increased glycolysis is needed when the ATP generation by oxidative phosphorylation is blocked. The liver FBPase also showed significant differences in the kinetic parameters between the control and anoxic turtles. The K_m_ FBP was 2.2-fold higher in the anoxic turtles as compared to the controls (Table 2). This represents a significant decrease in the substrate affinity and is indicative of a less active FBPase during anoxia. The results obtained here correspond well with the findings of previous studies. For example, Warren and Jackson [3] reported that, under anoxia, gluconeogenesis is suppressed due to high energy costs. Under low oxygen conditions, the reprioritization of the available ATP is essential to sustain the key cellular processes and promote pro-survival pathways. Therefore, gluconeogenesis might not be important during the early hours of anoxia; however, it would be required during the recovery from anoxia when large amounts of lactate must be cleared [6,11,12].

The activity of FBPase was negatively modulated by the allosteric inhibitors AMP and ADP [29]. Table 2 shows the I_50_ values for ADP and AMP. I_50_ means the concentration of an inhibitor that is needed to reduce enzyme activity by 50%. So, the current data represent that it takes an AMP concentration of 11.8 µM to reduce the FBPase activity of the control enzyme by 50% but significantly less (8.3 µM) to reduce the activity of anoxic FBPase. This shows that ADP is a stronger inhibitor of the anoxic form of FBPase as compared with the aerobic form. So, if the AMP or ADP levels rise in anoxia (which they do), their higher levels will have a stronger effect on the activity of anoxic FBPase than on aerobic FBPase. Such differences in the power of the inhibitors to control an enzyme are often due to PTMs that change the conformation of the enzyme protein and thereby allow greater or lesser access to the enzyme by substrate or allosteric modulators.

Previous studies of the liver FBPase from the little brown bat (*Myotis lucifugus*) [30] and white muscle of freshwater rainbow trout (*Oncorhynchus mykiss*) [31] showed similar responses to ADP and AMP under stress conditions [30,32,33]. The net effect of the increased inhibition of anoxic FBPase in liver when compared to the controls would result in the suppression of enzymatic activity under stress conditions. Furthermore, the increased inhibition by ADP and AMP indicates that activity is closely regulated by adenylate energy status. FBPase being the rate-limiting enzyme of gluconeogenesis, the adenylate levels would be important in controlling the rate of gluconeogenesis from the substrates, including glycerol-3-phosphate (resulting from triglyceride oxidation) or amino acids (from protein degradation) or lactate from glycogen catabolism [34]. This suggests that the sensitivity to AMP and ADP was increased under the anoxic condition so that the activities of energy-expensive pathways, such as gluconeogenesis, would be suppressed to prevent fruitless carbon cycling and wasteful ATP hydrolysis under energy-limiting conditions. Further analysis of the liver FBPase activity also showed its sensitivity to ATP.Mg, the enzyme from anoxic turtles showing a ~6-fold increase in its sensitivity to ATP as determined by a significantly decreased *K_a_* value of the anoxic liver FBPase (Table 2). A previous study on anoxic turtle liver showed a decrease in glycolytic intermediates during anoxia and the inhibition of glycolysis at the phosphofructokinase step to facilitate glycogenolysis [33]. The inhibition of glycolysis is also correlated with metabolic rate depression. Inhibiting both the glycolytic rate (via PFK) and gluconeogenesis rate (via FBPase) would reduce the overall carbohydrate metabolism, both substantially and in a co-ordinated fashion. Therefore, the increase in the affinity of ATP.Mg in the anoxic liver FBPase does not necessarily indicate an active anoxic FBPase. In fact, a study on turtle liver FBPase showed that anoxia had no effect on the total activity of FBPase, suggesting that FBPase activity might be regulated at the post-translational level [35]. The current results suggest that the initiation of gluconeogenesis would be more energy efficient under aerobic conditions to break down the lactate accumulated during anoxia. As such, perhaps FBPase activity is required during the early stages of recovery; therefore, increased sensitivity towards ATP.Mg could hasten gluconeogenesis during the early hours of recovery.

The additional analysis of liver FBPase was done to assess the effects of other putative modulators of enzyme activity. Phosphoenolpyruvate (PEP) was found to be the activator of liver FBPase. The Ka values significantly increased in the presence of PEP in the anoxic liver compared to the control values (Table 2). Flux through glycolysis is rerouted at the level of PEP. PEP concentration could co-ordinate the regulation of FBPase and Phosphofructokinase (PFK) in one of the two ways: 1. gluconeogenesis is favored when the cell is rich in biosynthetic precursors and ATP, or 2. high concentrations of PEP can activate FBPase. It is apparent from previous work that the short-term control of hepatic gluconeogenesis by glucagon and insulin may be of physiological importance [36]. Studies on rat liver suggest that the carboxylation of pyruvate, the pyruvate kinase reaction, and the fructose 6 phosphate/fructose 1,6 bisphosphate cycle have been forwarded as probable regulatory sites of the acute hormonal control of gluconeogenesis [37,38]. Pyruvate kinase is relatively the most important locus of endocrine control [39,40]. It is well established that glucagon exerts its effect at this level by stimulating the cyclic AMP-dependent phosphorylation of pyruvate kinase [41], which decreases PK affinity for PEP, thereby decreasing flux through pyruvate kinase despite increased PEP to pyruvate concentration ratios during gluconeogenesis. As a consequence, futile cycling between PEP and pyruvate is reduced and gluconeogenesis is effectively promoted [42]. A decrease in the affinity of PEP towards the anoxic liver FBPase may suggest a decrease in the activity of the enzyme towards PEP. The results obtained here further suggest another physiological function of FBPase, that is to prevent futile cycling during gluconeogenesis. Taken together, changes in the substrate affinity and susceptibility to allosteric inhibitors and activators may be co-ordinately regulated to result in an overall decrease in FBPase function as part of anoxia-induced hypometabolism while still maintaining sufficient activity to support basal ATP production by anaerobic glycolysis.

From the data obtained on kinetic parameters, it was apparent that structural differences might exist between the control and anoxic forms of FBPase. The potential reasons for the structural differences could be PTMs, especially phosphorylation. Protein phosphorylation is known to be a recurrent way to reversibly regulate enzyme activity in response to environmental stress in many animals [43]. The immunoblotting showed a significant increase of 1.4-fold in the phosphorylation of threonine residues on FBPase from the liver of anoxic turtles as compared with the enzyme from the control turtles (Figure 3). Threonine phosphorylation has been reported to suppress enzyme activities during anoxia and other forms of hypometabolism. For example, previous studies on the marine mollusk, *Busycon canaliculatum* [44], and the red-eared slider [45] showed a correlation between increased pyruvate kinase phosphorylation and changes in enzyme properties that would suppress enzyme function. Furthermore, based on an electrophoretic analysis of [^32^P] incorporation, it was suggested that O-phosphothreonine residue(s) were produced under anoxia [46]. By contrast, the post-translational modification by SUMOylation and ubiquitination decreased significantly to 22% and 73% of the control values during anoxia. In general, ubiquitination leads to protein degradation [47]. Furthermore, it has been shown that reduced ubiquitination stabilizes oxidized proteins [48,49]. Hence, under anoxic conditions, a decrease in the ubiquitination levels can suggest an increase in the enzyme stability in the energy-limited anoxic state. Similar to ubiquitination, the function of protein SUMOylation is less well understood. One study showed that SUMOylation contributed to the activation of the pro-survival pathways during stress [50]. A study of the muscle lactate dehydrogenase from *T. s. elegans* showed reduced ubiquitination and SUMOylation of the enzyme [51] in response to anoxia, which further supports our findings for liver FBPase. While the consequences of the ubiquitination and SUMOylation of turtle FBPase are still unknown, previous research has identified these PTMs as being crucial regulators of enzyme activity, protein–protein interactions, and protein stability [52].

This evidence of altered FBPase function under anoxia is in accordance with comparable findings of anoxia-responsive changes in the properties of other enzymes of carbohydrate metabolism in turtle organs, including glycogen phosphorylase, phosphofructokinase, and pyruvate kinase [7,8]. The anoxia-induced suppression of key energy-expensive processes (e.g., ion pumping, protein translation, urea synthesis, etc.) in isolated turtle hepatocytes [53] is also well documented. In these and other cases, including altered transcription factor responses under anoxia, the mechanism involved appears to be covalent modification by reversible protein phosphorylation [54,55]. These modifications at the binding sites of the enzyme can affect enzyme activity and sensitivity towards effectors under different conditions. The exposure to anoxia and long-term survival in an anoxic state requires regulatory mechanisms that enable a primary focus on only pro-survival processes under hypometabolic conditions. Being reversible processes, PTMs provide an economical solution to alter enzyme activity as opposed to more radical energy-expensive solutions, such as the proteolysis/resynthesis of enzymes over the anoxia/reoxygenation cycle. Changes in the enzyme activities and catalytic properties due to PTMs in response to environmental stresses have been observed in multiple previous studies conducted on both vertebrate and invertebrate species [23,45,56,57,58]. Considering the above results, and previous studies, it can be said that PTMs are an important factor behind the differential activity of an enzyme under aerobic versus anoxic conditions.

## 5. Conclusions

The data presented here show that turtle liver FBPase undergoes several significant changes in its functional and structural properties in response to anoxia. Differences in PTMs were linked with changes in the kinetic properties of the FBPase. In addition to that, the anoxic FBPase showed lower Vmax and decreased affinity for the substrate as compared to the control liver FBPase. The current study asserts post-translational control over gluconeogenesis, which further upholds the previous studies of metabolic responses to oxygen deprivation by anoxia-tolerant species. It also provides new insights regarding the metabolic reorganization that freshwater turtles undergo to cope with anoxia.

## Figures and Tables

**Table 1:** Purification of FBPase from liver from anoxic *T. s. elegans*. Two steps were used: (a) Phosphocellulose column chromatography with elution using a 0–1 M KCl gradient with 2.5 mM FBP added, and (b) CM column chromatography with elution using a 0–1 M KCl gradient.

Purification Step	Total Protein (mg)	Total Activity (mU)	Specific Activity (mU/mg)	Fold Purification	% Yield
Crude	21.07	94.25	4.47	---	---
PC	2.87	48.75	16.97	3.80	51.72
CM	0.59	23.4	39.13	8.75	24.82

**Table 2:** Kinetic parameters for the forward reaction of purified *T. s. elegans* liver FBPase, comparing control versus anoxia conditions. V_max_ and K_m_ values were determined at subsaturating FBP concentration (0.24 µM). Data are means ± SEM, n = Assays were performed at 22 °C. ‘*’ indicates that values for anoxic FBPase are significantly different from the control (*n* = 3–4) as determined by Student’s *t*-test *p* < 0.05.

Substrate	Enzyme Parameters	Control	Anoxic
	V_max_ (U/mg)	0.34 ± 0.010	0.25 ± 0.00 *
FBP(µM)	K_m_	26.9 ± 3.0	59.8 ± 5.2 *
ADP (µM)	I_50_	11.8 ± 0.3	8.3 ± 0.6 *
AMP (µM)	I_50_	6.1 ± 0.1	3.6 ± 0.3 *
Alanine (µM)	K_a_	2.85 ± 0.24	3.3 ± 0.1
	Fold Activation	2.5 ± 0.08	2.9 ± 0.03 *
PEP (µM)	K_a_	0.33 ± 0.09	3.3 ± 0.2 *
	Fold Activation	2.5 ± 0.05	3.0 ± 0.03 *
ATP.Mg (µM)	K_a_	144.5 ± 7.0	25.0 ± 0.9 *
	Fold Activation	1.9 ± 0.1	4.0 ± 0.02 *

## Data Availability

The data that support the findings of this study are available from the corresponding author upon reasonable request.

## Abbreviations

FBPase	Fructose-1,6-bisphosphatase
FBP	Fructose-1,6-bisphosphate
PFK	phosphofructokinase
PEP	Phosphoenolpyruvate
